# Supplementary material for: Characterizing and Eliminating the Inbreeding Load
Source: Vet Sci. 2023 Dec 22;11(1):8. doi: 10.3390/vetsci11010008 (PMC10819885; doi:10.3390/vetsci11010008)
Supplement: Supplementary file 1 [file vetsci-11-00008-s001.zip › vetsci-2716253-supplementary.pdf]

Supplement Table S1: Inbreeding load related parameters

Studies	Concept	Sign	Definition	Formula	Software
Wright [1]<br>Suwanlee at al. [2]<br>Boichard [3]<br>Gutiérrez et al. [4]<br>Coster [5][6]	Inbreedin g coefficient	F	The probability that the two alleles at any locus in an individual are identical by descent	$\mathrm{F}_x = \sum \left(\frac{1}{2}\right)^{n+n'+1} (1+f_a)$	PEDIG<br>ENDOG<br>Package "pedigree " in R
Ballou [1][7]<br>Baumung et al. [8]<br>00D,oekes et al. [2][6]	Ancestral inbreeding coefficient	F_{BAL}	The probability that any allele in an individual has been autozygous in previous generations at least once	$F_{Bal(x)} = \frac{\begin{bmatrix} F_{Bal(s)} + \left(1 - F_{Bal(s)}\right) * F_{W(s)} + \\ F_{Bal(d)} + \left(1 - F_{Bal(d)}\right) * F_{W(d)} \end{bmatrix}}{2}$	GRAIN
Kalinowski et al. [9]<br>Baumung et al. [8]<br>Doekes et al. [6]	Ancestral inbreeding coefficient	F_{KAL}	The probability that any allele in an individual is currently autozygous and has been autozygous in previous generations at least once.	Equation was not given	GRAIN
Kalinowski et al. [9]<br>Baumung et al. [8]<br>Doekes et al. [6]	Kalinowsk i "new" inbreeding coefficient	F_{NEW}	The probability that any allele in an individual is autozygous for the first time.	$F_{New(x)} = F_{W(x)} - F_{Kal(x)}$	GRAIN

Baumung et al. [8]<br>Doekes et al. [6]	Ancestral inbreeding coefficient (Ancestral history coefficient)	A_{HC}	The number that tells how many times during pedigree segregation (gene dropping) a randomly taken allele has been in IBD status	Equation was not given	GRAIN
Slatis [1]<br>Kennedy et al. [10]	Likelihood of genetic death	ll	The likelihood of genetic death (ie. all founders carry a recessive lethal gene)	Equation was not given	R script
Hinrichs et al. [11]	Ancestral inbreeding coefficient	F_{OLD}	The inbreeding occurring further back in the population history ("Old" inbreeding)	$F_{i,old}(t,u) = \frac{F_i(0,u) - F_i(t,u)}{1 - F_i(t,u)}$	PEDIG
Hinrichs et al. [11]	New inbreeding coefficient	F_{NEW_H}	The inbreeding occurring in recent generations ("New" inbreeding)	$F_{i,new}(t,u) = F_i(0,u) - F_{i,old}(t,u)$	PEDIG
López-Cortegano et al. [12]<br>García-Dorado et al. [13]	Purging coefficient	d	The part of deleterious effect that is exposed to genetic purging due to inbreeding	d = s(1/2–h)	PURGd
López-Cortegano et al. [12]<br>López-Cortegano et al. [14]	Purged inbreeding coefficient	g	The inbreeding coefficient (F) adjusted by the deleterious frequency that is expected by considering purging	$\hat{g} = \frac{1 - 2d}{1 + 2d(2N - 1)}$	PurgeR

Kardos et al. [15]	Genomic inbreeding coefficient	F_{ROH}	The proportion of the autosomal genome, in which autozygosity is derived from the assumption that very long stretches of homozygosity (ROH) can only result from inbreeding	$F_{ROH} = \Sigma L_{ROH}/L_{AUTOSOME}$	PLINK version 1.07
Gulisija and Crow [14]	Expressed opportunity for purging	O_E	The expressed opportunity for purging is the potential for reduction in expressed load in the present generation as a consequence of having inbred ancestors	$O_{Ei} = \sum 2F_{i(j)}F_j$	PurgeR
Morton et al. [16] Hoeck et al. [17]	The number of lethal equivalents	B	The number of deleterious genes per gamete that, when combined in a homozygous state, would result in the death of an individual	$S_F = e^{-A-BF}$	R script

- F_x is the conventional inbreeding coefficient of the individual x, f_a is the inbreeding coefficient of the common ancestors, n and n′ are the number of generations from sire and dam respectively to the ancestor of individual x.

- $F_{Bal(x)}$ is the ancestral inbreeding coefficient for an individual *(x)* with the subscripts (s) and (d) representing for the sire and dam, respectively.

- $F_{i,old}\ (t, u)$ is the old inbreeding coefficient of individual i in generation u with respect to a base generation at generation t.

- $F_{i,new}\ (t, u)$ is the new inbreeding coefficient of individual i in generation u with respect to a base generation at generation t.

- s is the selection coefficient against the homozygote and h is the dominance coefficient

- $\hat{g}$ is the predicted purged inbreeding coefficient based on the purging coefficient d and effective population size N

- ΣL_{ROH} is the total length of all ROH according to a priory specified threshold of succeeding number of homozygotes SNP obtained from the chip arrays, and $L_{AUTOSOME}$ is the specified length of the autosomal genome covered by SNP in chip

- O_{Ei}, is the probability that an allele, autozygous in *I*, $F_{i(j)}$ is the probability of an allele in *I* being derived from an allele in *j* and being autozygous in *i* ;

- S_F is the probability of survival of individuals with an inbreeding coefficient of ***F***, e^{-A} is the survival in a non-inbred random mating population (F = 0) and B is haploid lethal equivalentsts;

References:

1. Wright, S. Coefficients of inbreeding and relationship. *Am. Nat*. **1922**, *56*, 330–338. https://doi.org/10.1086/279872.
2. Suwanlee, S.; Baumung, R.; Sölkner, J.; Curik, I. Evaluation of ancestral inbreeding coefficients: ballou's formula versus gene dropping. *Conserv Genet*. **2007**, *8*, 489–495. https://doi.org/10.1007/s10592-006-9187-9.
3. Boichard, D. PEDIG: A fortran package for pedigree analysis suited for large populations. In Proceedings of the 7th World Congress on Genetics Applied to Livestock Production, Montpellier, France, 19–23 August **2002**; pp. 525–528.
4. Coster, A.; Coster, M.A. Package 'Pedigree'. R Package Version 1.4. 2010. Available online: https: //cran.r-project.org/web/packages/pedigree/index.html (accessed on 21 October 2023).
5. Gutiérrez, J.P.; Goyache, F. A note on ENDOG: A computer program for analysing pedigree information. *J. Anim. Breed. Genet*. **2005**, *122*, 172–176. https://doi.org/10.1111/j.1439-0388.2005.00512.x
6. Doekes, H.P.; Curik, I.; Nagy, I.; Farkas, J.; Kövér, G.; Windig, J.J. Revised calculation of Kalinowski's ancestral and new inbreeding coefficients. *Diversity* **2020**, *12*, 155. https://doi.org/10.3390/d12040155.
7. Ballou, J.D. Ancestral inbreeding only minimally affects inbreeding depression in mammalian populations. J. Hered. 1997, 88, 169–178. https://doi.org/10.1093/oxfordjournals.jhered.a023085
8. Baumung, R.; Farkas, J.; Boichard, D.; Mészáros, G.; Sölkner, J.; Curik, I. GRAIN: A computer program to calculate ancestral and partial inbreeding coefficients using a gene dropping approach. *J. Anim. Breed. Genet*. **2015**, *132*, 100–108. https://doi.org/10.1111/jbg.12145.
9. Templeton, A.R.; Read, B. Elimination of inbreeding depression from a captive population of Speke's gazelle: Validity of the original statistical analysis and confirmation by permutation testing. *Zoo Biol*. **1998**, *17*, 77–94.
10. Kennedy, E.S.; Grueber, C.E.; Duncan, R.P.; Jamieson, I.G. Severe inbreeding depression and no evidence of purging in an extremely inbred wild species—The Chatham Island black robin. Evolution 2014, 68, 987–995. https://doi.org/10.1111/evo.12315
11. Hinrichs, D.; Meuwissen, T.H.E.; Ødegard, J.; Holt, M.; Vangen, O.; Woolliams, J.A.; Analysis of inbreeding depression in the first litter size of mice in a long-term selection experiment with respect to the age of the inbreeding. *Heredity* **2007**, *99*, 81–88. https://doi.org/10.1038/sj.hdy.6800968.
12. López-Cortegano, E.; Moreno, E.; García-Dorado, A. Genetic purging in captive endangered ungulates with extremely low effective population sizes. *Heredity* **2021**, *127*, 433–442. https://doi.org/10.1038/s41437-021-00473-2 4.
13. García-Dorado, A.; Wang, J.; López-Cortegano, E. Predictive model and software for inbreeding-purging analysis of pedigreed populations. *G3: Genes Genomes Genet.* **2016**, *6*, 3593–3601. https://doi.org/10.1534/g3.116.032425.

14. López-Cortegano, E. PurgeR: Inbreeding and purging in pedigreed populations. *Bioinformatics* **2022**, 38, 564–565. https://doi.org/10.1093/bioinformatics/btab599.
15. Kardos, M.; Luikart, G.; Allendorf, F.W. Measuring individual inbreeding in the age of genomics: Marker based measures are better than pedigrees. *Heredity* **2015**, *115*, 63–72. https://doi.org/10.1038/hdy.2015.17.
16. Morton, N.E.; Crow, J.F.; Muller, H.J. An estimate of the mutational damage in man from data on consanguineous marriages. *Proc. Natl. Acad. Sci. USA* **1956**, *42*, 855–863. https://doi.org/10.1073/pnas.42.11.855.
17. Hoeck, P.E.A.; Wolak, M.E.; Switzer, R.A.; Kuehler, C.M.; Lieberman, A.A. Effects of inbreeding and parental incubation on captive breeding success in Hawaiian crows. *Biol. Conserv.* **2015**, *184*, 357–364. https://doi.org/10.1016/j.biocon.2015.02.011.
